# Release Pattern of Light Aromatic Hydrocarbons during the Biomass Roasting Process

**DOI:** 10.3390/molecules29061188

**Published:** 2024-03-07

**Authors:** Yaying Zhao, Yuqing Yan, Yuhang Jiang, Yang Cao, Zhuozhi Wang, Jiapeng Li, Chenshuai Yan, Danya Wang, Lu Yuan, Guangbo Zhao

**Affiliations:** 1 School of Energy and Power Engineering, Northeast Electric Power University, Jilin 132012, China; 20102341@neepu.edu.cn (Y.Z.); yy6738q@163.com (Y.Y.); hang2023678@163.com (Y.J.); 13213679895@163.com (J.L.); ycs@neepu.edu.cn (C.Y.); wang13514443416@163.com (D.W.); 18340374389@163.com (L.Y.); 2 School of Chemical Engineering and Technology, Hebei University of Technology, Tianjin 300401, China; 3 School of Energy Science and Engineering, Harbin Institute of Technology, Harbin 150001, China; zhaogb@hit.edu.cn

**Keywords:** biomass, baking, reaction conditions, catalysts, monocyclic aromatic hydrocarbons

## Abstract

Roasting is an important step in the pretreatment of biomass upgrading. Roasting can improve the fuel quality of biomass, reduce the O/C and H/C ratios in the biomass, and provide the biomass with a fuel quality comparable to that of lignite. Therefore, studying the structure and component evolution laws during biomass roasting treatment is important for the rational and efficient utilization of biomass. When the roasting temperature is 200–300 °C, the cellulose and hemicellulose in the biomass undergo a depolymerization reaction, releasing many monocyclic aromatic hydrocarbons with high reactivity. The proportion of monocyclic aromatic hydrocarbons in biomass roasting products can be effectively regulated by controlling the reaction temperature, residence time, catalyst, baking atmosphere, and other factors in the biomass roasting process. This paper focuses on the dissociation law of organic components in the pretreatment process of biomass roasting.

## 1. Introduction

Currently, energy consumption is highly dependent on traditional fossil fuels, with increased loads of carbon dioxide (CO_2_) and other greenhouse gases (GHGs). Traditional fossil fuels are causing widespread damage to the atmosphere and contributing to catastrophic global climate change [1]. The State Council issued a Circular on the State Council’s Action Program on Peak Carbon by 2030 in 2021, which states that new progress has been made in the research, development, popularization, and application of green and low-carbon technologies; that green production and lifestyles have been universally implemented; and that policy systems conducive to green, low-carbon, and recycling development have been further improved. To achieve a “carbon peak” and become “carbon neutral”, we should gradually reduce the consumption of fossil energy and, at the same time, effectively increase the proportion of renewable energy consumption [2]. With the development of national economy, energy consumption is gradually increasing and facing greater environmental pressure. As an important part of energy, biomass reserves are second only to the three major fossil energy sources. They have characteristics of renewability, low pollution, carbon neutrality, rich reserves and broad development prospects. They can not only relieve the energy pressure in China, but also reduce the carbon dioxide emissions of fossil energy, and help to achieve the goal of “double carbon” [3]. With the development of the rural economy and the increase in farmers’ income, straw burned in the traditional way has become the object to be replaced, resulting in the increase of straw in abandoned fields year by year. The widespread burning of straw has caused serious air pollution. Therefore, low-carbon energy derived from biomass has been developed to reduce the dependence on fossil energy. Roasting refers to a heat treatment method in which the temperature of biomass is in a low-oxygen or oxygen-free atmosphere. Lignin will be partially decomposed in roasting, and phenols are the most important volatile products, in addition to a small amount of acetic acid, ethanol, formaldehyde, etc. Roasting is a process to upgrade waste lignocellulose (so not in competition with food), aiming towards multiple products such as chemicals, char, and also fuels. Light aromatic hydrocarbons, such as benzene, toluene, and xylene, are important raw materials in the production of various industrial products, such as plastics, resins, solvents, and pharmaceuticals. Biomass roasting not only removes the water content but also produces several derivatives. To cut costs, it is essential to make full use of roasted biomass as a by-product of the treatment process. Various pretreatment techniques may be improved and combined to overcome the limitations of a single method, resulting in synergies.

Previous studies have focused on the yield of monocyclic aromatic hydrocarbons under a single torrefaction condition. This paper reviews the release patterns of monocyclic aromatic hydrocarbons from biomass under different torrefaction conditions, and it guides subsequent experimental research. This study aims to optimize the production of monocyclic aromatic hydrocarbons after torrefaction through the synergistic effects of torrefaction temperature, torrefaction time, torrefaction atmosphere, and catalysts. Aromatic components replace the moisture in lignite, improve the energy density and combustion activity of lignite after drying, and further enhance the fuel quality and hydrophobic properties of lignite.

## 2. Macroscopic Effects of Baking Pretreatment on Biomass

Margareta [4] found that roasting improved the fuel quality of biomass, increased its hydrophobicity, and reduced the moisture retention capacity of the roasted samples. Che et al. [5] found that the high-level heating capacity of the roasted feedstock increased by 38.9%, comparable to that of lignite. Matt et al. [6] found that the grindability index of roasted corn cobs significantly increased, the bio-oil yield reached 51.7%, and the bio-coke yield was effectively increased. Wang [7] found that the energy consumption for grinding dry wood after roasting at 300 °C was 50% of that before roasting. Hwai [8] found that roasting effectively reduces the O/C ratio and moisture content in biomass, and a large amount of hemicellulose and cellulose depolymerization during roasting reduces the length of the fiber structure, which can effectively improve the biomass’s grindability. Chen [9] found that the large amount of dissociation or transformation of OH groups during roasting led to a decrease in the bonding ability of hydrogen bonds on the biomass surface, effectively reducing the moisture-binding ability of the roasted products. Many nonpolar unsaturated structures were formed due to the reconstruction of the chemical structure during roasting and, as a result, the roasted biomass was not easily biodegraded, even after storage for a long time. Therefore, roasting can effectively increase the energy density and hydrophobicity of biomass-based fuels and reduce the energy consumption of biomass milling.

## 3. Influence of Roasting Atmosphere on Biomass Refining Effect

**Figure 1:**
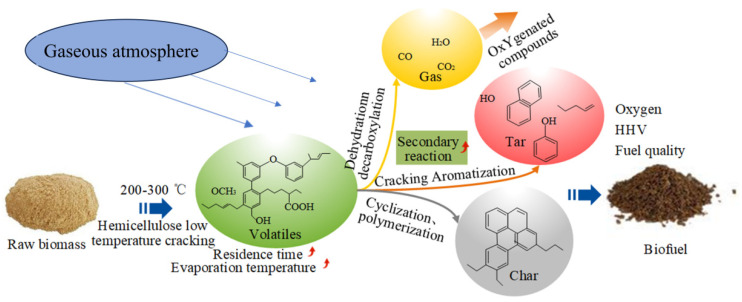
Torrefaction atmosphere [15].

Cahyanti [10] carried out constant-temperature baking experiments in a 0–21% oxygen concentration range and a 230–310 °C temperature range. The results showed that increasing the temperature could effectively enhance the baking reaction rate with a fixed oxygen concentration, and increasing the oxygen concentration could effectively enhance the baking rate with a fixed temperature. Shen [11] investigated the baking characteristics of hemicellulose (xylan-based) under different atmospheres (inert and oxidizing). The results showed that the CO and CO_2_ in the exhaust gas gradually increased with an increasing oxygen concentration. Chen [12] found that aerobic roasting could promote the dehydroxylation, decarbonylation, and decarboxylation of cellulose; reduce the initial temperature of cellulose pyrolysis and the maximum rate of heat weight loss; and increase the yield of pyrolytic char. The activation energy of cellulose pyrolysis after roasting gradually increased with the increasing oxygen concentration. Wang [13] found that the low-oxygen roasting of cedar wood chips increased their abradability, decreased the release of aldehydes and ketones, and significantly improved their hydrophobicity and storability. Brachi [14] studied the aerobic and anaerobic roasting of woody fir and non-woody olive pomace. The results showed that the fuel quality enhancement was more pronounced after the inert roasting of woody fir. Li [15] pretreated bamboo via roasting under a nitrogen atmosphere (Figure 1). The results showed that inert roasting improved the bamboo’s combustion characteristics.

## 4. Influence of Roasting Method on Biomass Fuel Quality

**Figure 2:**
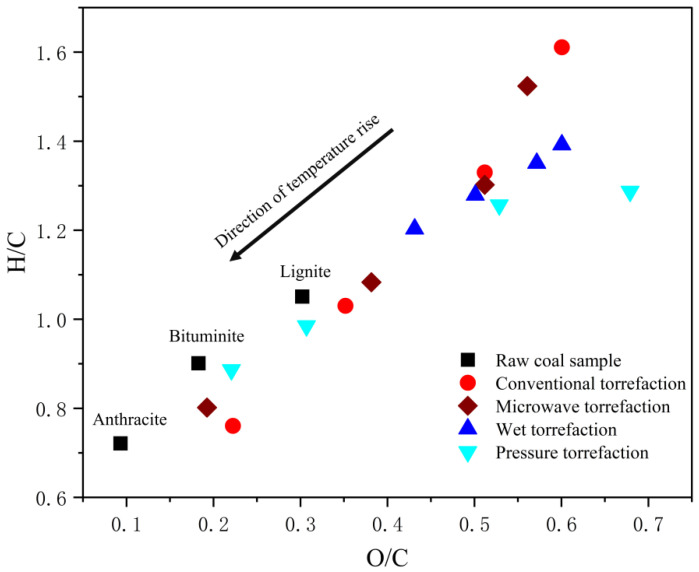
H/C-O/C two-phase diagram of torrefaction samples [24].

Medic [16] applied conventional roasting to increase the energy density and reduce the oxygen percentage of corn stover, which led to the conversion of oxygen in the structure to CO and CO_2_. Wang [17] investigated the use of microwave roasting for rice husk and bagasse and found that microwave roasting has more efficient heating and deoxidizing effects and is adaptable to the biomass particle size. Zhang [18] used wet roasting to pretreat rice husk and found that it not only promoted the generation of bio-oil but also removed some minerals from the biomass. Yuan [19] found that the roasting solution from wet roasting can be used to produce furfural and organic acids and promote the production of phenols. Gao [20] found that the pressurized roasting of biomass can efficiently promote dehydration and decarboxylation, and the effect of deoxygenation is higher than that of atmospheric pressure roasting. Moreover, pressurized roasting can significantly change the physical structure of the biomass, resulting in a richer pore structure. It also significantly promotes the release of volatile matter in the roasting process and optimizes the combustion characteristics of the biomass. Miura [21] discovered that conducting baking experiments under mechanical pressure can promote the cross-linking reaction of -OH groups in biomass, and the deoxygenation effect is better than that of atmospheric pressure baking. Sun’s [22] study found that roasting under atmospheric pressures promoted the autonomous deoxygenation of rice straw biomass. In Shan’s [23] study on the effect of pressure on deoxygenation during biomass roasting, the oxygen content of the biomass after pressurized roasting was reduced to 20%, which was much lower than that of biomass roasted at atmospheric pressure and was close to that of sub-bituminous coal. Gao [24] investigated the pressurized roasting of corn stover and analyzed the roasted bio-oil, finding that the yield of monocyclic aromatic hydrocarbons reached 3.85% at 230 °C under pressurized roasting (Figure 2). The temperature required for gas-pressurized roasting is usually approximately 40 °C lower than that for atmospheric roasting. Previous studies have found that the roasting method mostly affects the structure of the biomass, which can increase the biomass’s calorific value and biochar yield and improve the modified biomass’s hydrophobicity; however, it has less of an effect on the yields of the liquid oils and aromatic fractions.

## 5. Effect of Baking Temperature on the Evolution of Organic Fractions in Biomass

Zhong et al. [25] found that hemicellulose started to degrade at 220 °C and reached the maximum rate of weight loss at approximately 268 °C with a residue of approximately 20%; cellulose depolymerized in the range of 315–400 °C, leaving only an approximately 6.5% solid residue; and lignin remained at increasing roasting temperature. Niu [26] found that roasting at approximately 250 °C gave better roasting results, generating roasted samples with relatively lower moisture and greater energy density. Zheng [27] found that the best roasting temperature for corn stover was 290 °C, and too high a temperature reduced the yield of three-phase products and caused more energy loss. Wannapeera et al. [28] conducted roasting experiments on biomass from 200 °C to 275 °C and found that the total amount of liquid-phase products significantly increased; the relative phenolics content in the bio-oil increased from 16.42% to 22.05%, and the phenolics yield gradually increased with the increasing roasting temperature. Cao [29] conducted roasting treatments on bamboo at four temperatures, 210, 240, 270, and 300 °C, and analyzed the components of the roasting liquid. The content of furans and ketones increased from 29.04% and 16.42% to 31.42% and 22.05%, respectively, while the content of aldehydes decreased from 8.26% to 6.18%.d with a 3.900% solid residue at 540 °C and formed a large amount of biochar.Simonic [30] found that the optimal roasting temperature for woody materials is approximately 260 °C, and the calorific value of the products can be maximized via roasting pretreatment at this temperature. Chen et al. [31] found that roasting pretreatment significantly affected the yield and quality of biochar and bio-oil. Valette [32] investigated the release pattern of condensables during the roasting of pine, ash, cumin, and wheat straw at 250, 280, and 300 °C. Carboxylic acids, alcohols, aldehydes, ketones, furans, and dehydrated sugars were the main organic parts of the condensables, and the yields of the condensable products were less affected by the roasting temperature. The baking temperature should not be too high. When the baking temperature is higher than 300 °C, most of the hemicellulose, part of the cellulose, and a small part of the lignin in the biomass begins to depolymerize, and the biomass undergoes significant weight loss, i.e., the biomass directly undergoes pyrolysis.

Potnuri [33] blended an adequate amount of KOH into wood chips, and roasting experiments were carried out at 125–175 °C. The results showed that the addition of KOH effectively increased the tar yield (57.1–59.6 wt%) and char yield (27.3–29 wt%) and that KOH acted as a regulator in the roasting process. In a study on the roasting of wood chips, Valizadeh [34] found that acids and phenols dominated the bio-oil at a roasting temperature of 300 °C and that increasing the roasting temperature decreased the yield of phenols in the bio-oil. In roasting experiments on hawthorn seeds, Zhao [35] found that when the roasting temperature was 250 °C, hemicellulose reacted to produce several phenolic compounds, furfural, acids, esters, and other substances. When the roasting temperature was elevated to 275 °C, the types of phenolics were drastically reduced from seven to three, and the phenolics mainly consisted of 2,6-dimethoxyphenol, with a content of more than 28%. When the roasting temperature was further elevated to 300 °C, the cellulose decomposition further increased the phenolics content in the bio-oil. The organic components in biomass gradually begin to decompose due to an increasing roasting temperature, and the roasting time indirectly affected the roasting results of the biomass. In roasting experiments on peanut shells, sawdust, and bamboo, Wen [36] found that extending the roasting time from 30 min to 90 min increased the fixed carbon content in the roasting products at a roasting temperature of 300 °C. Among these products, the fixed carbon content in sawdust and bamboo significantly varied during the roasting time of 90 min, but that of peanut shells steadily varied with the increase in the roasting time. The variation in the fixed carbon content was relatively stable with the increase in the roasting time. The content of volatile matter in the three biomasses significantly decreased with the increase in the baking time, but the effect of the baking time on the fixed carbon and volatile matter content was small compared with that of the baking temperature. The effect of the roasting temperature on the contents of components in the biomass was much greater than that of the residence time. The effect of the residence time was approximately 3–7 times lower than that of the temperature [37]. It is generally believed that the influence of the roasting conditions on the roasting effect follows the order of roasting temperature > residence time > roasting method > particle size of raw materials [38]. The roasting effect of biomass under different roasting temperature conditions is also different. The first stage, when the temperature is less than 200 °C, mainly involves the dehydration of the biomass. In the second stage, when the temperature is in the range of 200–300 °C, the decomposition of part of the hemicellulose mainly occurs. In the third stage, when the temperature is greater than 300 °C, the cellulose also begins to decompose. The yield of the bio-oil is directly proportional to the cellulose content in the biomass. The yield of biochar is directly proportional to the content of lignin in the biomass due to the stronger thermal stability of lignin.

## 6. Effect of Catalyst Baking on the Evolution of Biomass Organic Fractions

**Figure 3:**
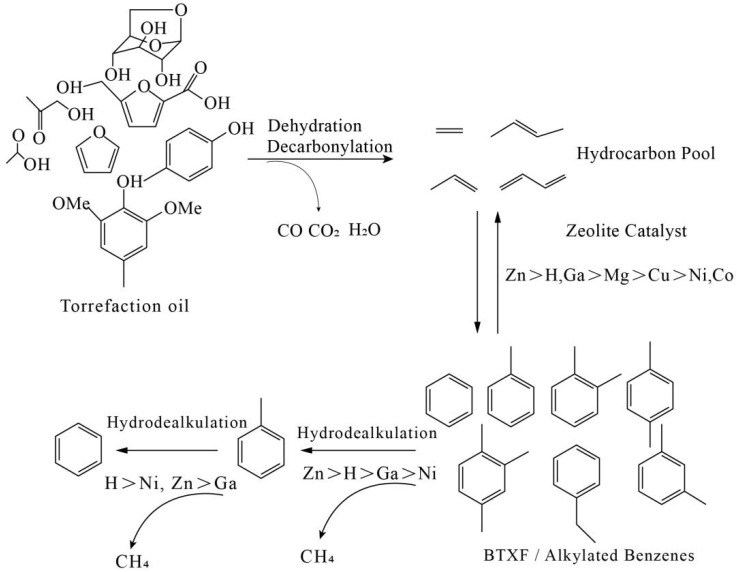
Catalytic mode of zeolite [48].

In addition to regulating the roasting temperature and time, adding a certain amount of a catalyst can effectively increase the yield of aromatic hydrocarbons in the biomass roasting process. The ZSM-5 molecular sieve is the most active and selective catalyst for producing aromatics from various biomass feedstocks due to its medium pore structure, high acidity, and high hydrothermal stability. During the catalytic process, intermediates (furans and hydroxides) diffuse into the zeolite’s catalytic sites and generate olefins and, ultimately, aromatics via a series of catalytic mechanisms, including deoxygenation, cleavage, and oligomerization, in the internal active sites. In a roasting experiment on cotton stalks, Chen [39] found that at the same roasting temperature, adding a certain amount of a catalyst caused the oxygen-containing organic molecules in the bio-oil to undergo decarbonylation, decarboxylation, dehydration, and zwitterionization reactions, and many oxygen-containing functional groups were removed, generating several aromatic hydrocarbons and other small molecules. The content of aromatic hydrocarbons in the product was the highest when the reaction temperature was 200 °C. Dai et al. [40] found that adding a certain amount of MgO in the biomass roasting process can effectively reduce the biochar yield, reduce the formation of acid in the bio-oil, and increase the aromatic hydrocarbon content in the bio-oil. Zheng [41] determined that some highly reactive oxygenated intermediates (e.g., furans, pyrans, and alkyl oxygen phenols) are generated at the beginning of the thermal depolymerization of biomass, and these intermediates tend to re-polymerize to form coke in the absence of a catalyst. The catalyst is key in the selective deoxygenation of oxygenated intermediates and in improving the bio-oil quality. Xiang [42] found that the selectivity of bio-oil aromatics was closely related to the interaction between zeolite’s acidity and porosity. Liang [43] found that introducing Al into SBA-15 enhanced the conversion of cellulose to furan, hemicellulose to furan and aromatics, and lignin to phenolics. Zhong [25] found that CaO, Al_2_O_3_, and ZnO can be used with ZSM-5 catalysts as new dual catalysts. The combination of CaO and ZSM-5 has a strong ability to remove carbonyl compounds and increase the formation of hydrocarbons (e.g., monocyclic aromatic hydrocarbons) while minimizing coke formation. Moreover, this combination can efficiently induce the conversion of wood chips to high-quality monocyclic aromatic hydrocarbons. Mullen et al. [44] investigated the effect of Ga on the entire reaction pathway from bio-oil vapor to aromatics. Two major competing reaction pathways for producing aromatics may occur after the dehydration of bio-oil vapors (e.g., anhydrous sugars) to olefins. In experiments with Ga-modified ZSM-5 (SAR = 30), Cheng [45] found that Ga had a superior dehydroxylation efficiency and could induce the aromatization of intermediates. Introducing a metal promotes deoxygenation mainly via decarboxylation and decarbonylation processes and generates more olefins for producing aromatics. Therefore, adding metal catalysts to graded ZSM-5, combining the advantages of the metal and graded structures, can improve the catalytic performance of biomass CFP and the production of aromatics [46]. Ni and Cu promote decarboxylation/decarbonylation reactions and aromatization, which, in turn, increase the aromatic yield [47]. However, higher Ni and Cu loading decreases the aromatic yield. It is not only metal ions that can increase the yield of aromatics. The aromatic product yield via HZSM-5 two-stage catalysis is approximately 2.66 times higher than that via single-stage catalysis [48] (Figure 3).

Zheng’s conclusions [49] are cited in Table 1 and Table 2. As shown in Table 1, different metal-modified ZSM-5 catalysts had different effects on the biomass aromatic yield and selectivity, and the use of metal-modified catalysts resulted in higher-quality aromatics. The bimetallic modification of ZSM-5 increased the stability of its structure due to changes in the catalyst’s acidity and strength, which, in turn, improved the yield and selectivity of aromatics. Ga modification of HZSM-5 mainly enlarged the catalyst’s pore size, increased the decarbonylation and olefin aromatization reaction rates, and improved the bio-oil yield and aromatics selectivity. Adding Zn, Ni, and Co changed the distribution of the acid sites, transforming the Brønsted acid into a Lewis acid, but did not change the total acid amount, thus reducing the carbon deposition and catalyst deactivation. Fe was effective in increasing the lignin and light olefin contents. The Fe/HZSM-5 catalyst promoted the generation of naphthalene and its derivatives.

The modulation of zeolites’ acidity is important for regulating the distribution of biomass pyrolysis products, which can be effectively achieved by introducing alkali metals into the zeolites. Co-, Fe-, Nb-, Ni-, and Zn-modified ZSM-5 catalysts all inhibited the production of PAHs in [50]. However, the selectivity for MAHs, especially BTX, varied with the loading metal in the metal-doped ZSM-5 catalysts due to different acidity distributions, as did the selectivity for specific bio-oil fractions.

Adding potassium to Ni/HZSM-5 decreases the total acidity and changes the ratio of acid sites, favoring the cracking of alkylated PAHs into lighter ones and reducing coke production [51]. Adding transition metals to zeolites also alters their acidity. Bio-oil’s aromatic selectivity depends on the zeolite’s acidity and porosity. In [42], the more acidic ZSM-5 (SAR 25) was less selective for phenols and oxygenated compounds, whereas sulfated lignin pyrolysis was more selective for bio-oil aromatics, indicating that the higher the acidity (the lower the Si/Al ratio), the higher the efficiency of the removal of the -OCH3 groups from the lignin, the greater the dehydration of the aliphatic -OH groups, and the higher cleavage of the aliphatic and etheric C-C bonds.

In [52], the Ga in Ga-modified ZSM-5 replaced the strong Brønsted–Lowry acid site with a weak Lewis acid site, which reduced the excessive cleavage of the biomass-derived intermediates. Furthermore, adding metallic nickel to the ZSM-5 facilitated the elimination of oxygen in the form of CO/CO2, thus leaving more available hydrocarbons and facilitating the conversion of phenols to aromatics. In [53], Ni-modified ZSM-5 exhibited higher aromatic yields and higher selectivity for MAHs than the parent ZSM-5 due to the enhanced dehydration at the Ni sites. Adding Ni also improved the water stabilization of the zeolite catalysts. Adding Zn to ZSM-5 partially converts the Brønsted–Lowry acid sites of the zeolite to Lewis acid sites, which stimulates the migration of h atoms via C-H activation and thus promotes the formation of hydrocarbons.

## 7. Summary

A review of the biomass roasting process revealed that the main factors influencing the evolutionary pattern of organic components in this process are the roasting temperature, catalyst, and roasting time.

1. The baking temperature selection is crucial. The baking temperature range should be between 200 °C and 300 °C. Excessively high temperatures will make the organic components of biomass rapidly decompose from baking to pyrolysis. At a baking temperature of 270 °C, biomass generally does not undergo significant decomposition and can produce some aromatic components. Moreover, the water is completely removed, and the oxygen content is further reduced by removing CO and CO. In addition, the complex cross-linking structure of the biomass is broken, and the methoxy in the more stable lignin is effectively removed.
2. The depolymerization of cellulose and hemicellulose occurs during biomass roasting. Adding catalysts such as ZSM-5 and zeolite during the roasting process enables the timely dehydroxylation and carboxylation of oxygenated intermediates in the produced bio-oil, thus converting the oxygenated intermediates to aromatics.
3. The baking method affects the degree of depolymerization of the organic components in the biomass. It has a lesser effect on the aromatic yield and a greater effect on the physical structure of the biomass and the biochar yield.
4. Other conditions have a minimal effect on the precipitation of aromatic components in the biomass baking product, which is negligible compared with the effects of the reaction temperature and catalyst.
5. Optimizing the baking temperature can improve the aromatic yield in the biomass baking stage. Moreover, using suitable catalysts can promote the conversion of oxygenated intermediates in the bio-oil to aromatics, and reasonable control of the baking temperature and catalyst type can effectively improve the yield of aromatic components.

This paper explored how roasting treatments affect the lightweight aromatics obtained from biomass, which must overcome certain obstacles before they are applied on a large scale. One of the most pertinent issues is the poor quality of biomasses, which have complicated cross-linking structures and low H/Cs, resulting in expensive aromatic conversion, low yields, and catalyst deactivation. Even though several by-products are currently created in biomass roasting, with further progress in technology and research, the mix of various treatments can be optimized under different conditions to generate unique effects. However, eliminating light aromatics is costly because of the limited technology for roasting and the complex product. Therefore, utilizing biomass and coal in synergy can have a mutually reinforcing effect.

Combining the roasting treatment of biomass with lignite drying and dewatering can passivate hydrophilicity. Lignite is characterized by a high water content (25–65 wt%), low energy density, and strong spontaneous combustion tendency, which severely limits the clean and efficient utilization of lignite and its safe storage and transportation. Dehydration pretreatment can remove a large amount of water from lignite and reduce its spontaneous combustion tendency. Aromatic components are difficult to separate from products generated in macroscopic quantities during biomass modification, and water-resistant organic matter, such as benzene, toluene, styrene, phenol, and naphthalene, comprise the main cracking products of biomass tar. The application of biomass-modified tail gas to dry high-water-content lignite can recover the tail gas waste heat and simultaneously weaken the moisture reabsorption capacity of the dried lignite by forming a water-blocking layer or passivating hydrophilic groups on the surface of lignite particles via tar and its cracking products. Therefore, applying biomass-modified flue gas rich in water-blocking organic matter to dry high-water-content lignite can weaken its moisture reabsorption capacity while realizing the low-temperature waste heat drying of the lignite from the flue gas. In addition, replacing the moisture in the lignite with aromatic components can improve the energy density, combustion reactivity, and fuel quality of the dried lignite. This has better development prospects and feasibility compared with the current biomass utilization technology.

## Figures and Tables

**Table 1:** Bio-aromatic hydrocarbons from catalytic pyrolysis of biomass compared with metal-modified HZSM-5 [49].

Catalyst	Feedstock	Ratio of Biomass to Catalyst	T/°C	Aromatic Content/%
HZSM-5	Prairie cordgrass	10:1	600	24.5
Co/HZSM-5	Prairie cordgrass	10:1	600	30.3
Mo/HZSM-5	Prairie cordgrass	10:1	600	26.7
MoO_3_/HZSM-5	Torrefied switchgrass	1:10	700	21.9
Mo_2_C/HZSM-5	Torrefied switchgrass	1:10	700	25.0
Fe/HZSM-5	switchgrass	1:10	550	17.0
P/HZSM-5	Rape straw	1:10	550	48.4
Zn/HZSM-5	Rape straw	1:10	550	44.7
Ti/HZSM-5	Rape straw	1:10	550	50.1
Co/HZSM-5	Jatropha residues	1:1	500	24.6
Ni/HZSM-5	Jatropha residues	1:1	500	20.9
Mo/HZSM-5	Jatropha residues	1:1	500	26.9
Ga/HZSM-5	Jatropha residues	1:1	500	27.6
Pd/HZSM-5	Jatropha residues	1:1	500	27.2
La/HZSM-5	Rape straw	-	500	49.9
Mg/HZSM-5	Woody	1:10	450	29.0
Cu/HZSM-5	Woody	1:10	450	31.0
Sn/HZSM-5	Woody	1:10	450	33.0

**Table 2:** Bio-aromatic hydrocarbons from catalytic pyrolysis of biomass compared with bimetallic modified HZSM-5 [49].

Catalyst	Feedstock	Ratio of Biomass to Catalyst	T/°C	Aromatic Content/%
Mo-Co/HZSM-5	Prairie cordgrass	10:1	600	41.1
Mo-Zn/HZSM-5	Torrefied switchgrass	1:10	700	39.3
Mo-Ag/HZSM-5	Torrefied switchgrass	1:10	700	23.8
Ga-Ni/HZSM-5	Debarked eucalyptus trunks	1:10	600	16.1
Zn-p/ZSM-5	Rape straw	1:10	500	74.6
Mo2N/HZSM-5	Pine wood	1:5	750	73.7
W2N/HZSM-5	Pine wood	1:5	750	43.4
Mo-P/HZSM-5	Pine wood	1:5	750	39.1
W-P/HZSM-5	Pine wood	1:5	750	60.6
Ce-Mo_2_N/HZSM-5	Pine wood	1:5	750	71.4
La-Mo_2_N/HZSM-5	Pine wood	1:5	750	17.6
Cr-Mo_2_N/HZSM-5	Pine wood	1:5	750	71.6
Cu-Mo_2_N/HZSM-5	Pine wood	1:5	750	61.1
Fe-Mo_2_N/HZSM-5	Pine wood	1:5	750	65.6
Mo-Co/HZSM-5	Lignite	1:1	600	80.7
Ni-Co/HZSM-5	Pine sawdust	1:1	600	75.2
Mo-Cu/HZSM-5	Sugarcane bagasse	1:10	550	36.5
Ni-Ce/HZSM-5	Pine wood	1:1	500	13.8
Zn-Ni/ZSM-5	Pine wood	1:2	500	38.5
Zn-Ga/ZSM-5	Pine wood	1:2	500	39.3
Zn-Co/ZSM-5	Pine wood	1:2	500	79.1
Ni-Cu/HZSM-5	Pine wood	1:2	450	46.5

## Data Availability

Not applicable.
